# Enhancing Adult Autism Diagnostic Pathways: The Role of Clinical Triage in Efficient Service Provision

**DOI:** 10.3390/jcm14092933

**Published:** 2025-04-24

**Authors:** Marios Adamou, Sarah L. Jones, Tim Fullen, Bronwen Alty, Jennifer Ward, Joanne Nixon Mills

**Affiliations:** 1 School of Human & Health Sciences, University of Huddersfield, Huddersfield HD1 3DH, UK; m.adamou@hud.ac.uk; 2 South West Yorkshire Partnership NHS Foundation Trust, Wakefield WF1 3SP, UK; tim.fullen@swyt.nhs.uk (T.F.); bronwen.alty@swyt.nhs.uk (B.A.); jennifer.ward@swyt.nhs.uk (J.W.); joanne.nixonmills@swyt.nhs.uk (J.N.M.)

**Keywords:** autism, adult autism, autism diagnosis, adult autism diagnosis

## Abstract

**Background**: Autism spectrum disorder (ASD) is a lifelong neurodevelopmental condition affecting 1.1% of adults. The increasing incidence of ASD has led to pressurised diagnostic services. **Objective**: We aimed to determine the number needed to harm (NNH) of criteria-informed triage assessment in an adult autism diagnostic service in the UK. **Methods**: The study was conducted at a specialist adult Autism Service in West Yorkshire, UK, from November 2021 to August 2022. All eligible referrals were accepted, with criteria requiring service users to be over 18 years old and without an intellectual disability. The evaluation consisted of 60 cases. **Results**: None of the evaluation cases resulted in a clinical diagnosis of ASD, yielding an infinite number needed to harm (NNH), demonstrating that every case benefited from the triage process without significant risk of harm. **Conclusions**: Triage enables services to gather comprehensive information about individual presentations and clinical needs, facilitating informed decision-making and better service utilisation. The evaluation demonstrates the safety and effectiveness of the triage process, with directions for further research discussed.

## 1. Introduction

Difficulties in social–emotional reciprocity and rigid or repetitive behaviours characterise autism spectrum disorder (ASD) [1]. ASD is estimated to be present in 1.1% of adults. ASD is often diagnosed in childhood and is a lifelong condition [2]. At present, there are no specific biomarkers; therefore, diagnosis is based on behavioural observations [3]. ASD populations often suffer from poor physical and mental health compared with non-ASD groups due to difficulties accessing healthcare [4], and it is estimated that difficulties in gaining employment affect 90% of the ASD population [5].

While we are still learning about the aetiology of ASD, we know more about ASD and how to respond to it than ever before [6]. It is suggested that for every three diagnosed cases, two cases remain undiagnosed. There is often particular difficulty in diagnosing ASD with lower support needs, as it is often not identifiable until adolescence or adulthood [7]. Additionally, individuals with ASD can often be misdiagnosed with other mental health conditions, such as depression, anxiety, personality disorders, or other disorders [5].

Without the robust identification and assessment of ASD, inadequate care is the result. Provisions for children are well established; however, the same cannot be said for adult services [5]. It has been suggested that healthcare services fail in a particular cohort, namely, adults with ASD who have an intelligence quotient of >70 and do not have mental illnesses. This cohort may be missed by both learning disability services and mental health services [8], a concerning assessment that adult ASD services must be sensitive to.

Between 1998 and 2018, the incidence of ASD increased by 787% in the UK [9]. Notably, the increase has been greater for females, and there has also been an increase in the reporting of ‘lower need’ cases. However, this growth is unlikely to be due to prevalence per se; it is more likely due to increased reporting and better access to diagnostic services [9]. Similarly, an Australian study reported a steady increase over the years, with a 200% increase in ASD diagnoses, despite a 5% decrease in the population. Furthermore, the gender ratio changed from 8:1 for boys to 3.5:2 from the 1980s to the 1990s [10]. While the prevalence of ASD continues to grow exponentially, there is an overwhelming demand for services [11]. Referrals to diagnostic services have greatly increased in recent years, and, as a result, services are under great pressure and are often struggling to keep up with demand [6,12].

Notably, the increased use of social media platforms has increased awareness of ASD, with #autism claiming over 11.5 billion views [13]. Social media allows health communities to connect, often sharing lived experiences [14]. While this has many benefits for health communities, such as reducing stigma and providing access to peer support, there is also concern surrounding the validity of the content and the creators. For example, a recent study revealed that of the top 113 videos available on social media via #autism, only 27% included accurate information, whereas 41% of the information was deemed inaccurate, and 32% was believed to be overgeneralised [13]. An additional concern is the use of symptom checkers and self-diagnostic tools available online; whereas there is no evidence of increased self-diagnosis, there is a suggestion that there is an increase in self-triage [15]. Naturally, it can be theorised that the use of digital platforms has been a factor in the recent increase in referrals for diagnostic assessment, putting greater pressure on an already-stretched system.

In terms of access to services, ASD populations are vulnerable to healthcare inequalities. This population has reported barriers to healthcare, dissatisfaction with diagnostic services, and general negative experiences with ASD services [16,17,18,19,20]. A cross-sectional study revealed that 80% of ASD service users reported difficulty accessing GP services. The reasons included deciding whether visiting the GP was appropriate for their needs, difficulty making telephone calls to make appointments, not feeling understood, difficulty communicating their needs, and struggles with the waiting room environment [4]. A qualitative study in Australia revealed that patients described their personal journey of the diagnostic process as ‘difficult’ and ‘time-consuming’ [21]. Secondary services must be better equipped to accommodate ASD service users and reduce wait times, especially considering that a UK study reported that 40% of service users were dissatisfied with their experience of the diagnostic process [22].

Acknowledging the experiences of service users is necessary to drive change and adapt services to meet needs. However, the experiences of healthcare professionals are also an important consideration. In a study of 116 multidisciplinary professionals working in ASD services, when asked about their experience of patient accessibility and the diagnostic process, 40% reported that they believed that services were unable to provide service users with timely assessments. They also voiced concern regarding the validity of standardised tools used for assessment, a well-documented concern [23,24,25,26], yet one beyond the scope of this discussion.

ASD diagnostic pathways have specific challenges. Specifically, the limited number of specialist workers, lengthy waiting lists due to increased demand, and complex multidisciplinary evaluations are not economically favourable and have a disproportionately negative impact on service users and families [2]. Wieckowski, Zuckerman [2] noted that in the United States, this is of great concern because services are adopting new approaches to ASD pathways (but they are as yet unevaluated). Similar concerns have been noted in the UK, with a recent debate concerning how diagnostic pathways should be designed to meet the recent influx in demand [6], including the proposal that those seeking diagnosis could be seen by specialist autism support workers rather than by clinicians in the first instance to ease the ever-increasing pressure on services [27].

In the UK, the common route (but not an exclusive one) for accessing diagnostic services is through National Health Service (NHS) GP services. GPs are the so-called ‘gatekeepers’ of secondary services, such as specialist ASD diagnostic services, to which they can refer if appropriate. Operational guidance, alongside the national framework for ASD assessment pathways provided by NHS England, provides principles that guide the delivery of an ASD assessment pathway. This is to ensure the inclusion of standardised key components for ASD assessment, such as the screening and triage process [28]. The guidance aims to provide services with direction to reduce health inequalities and improve outcomes in ASD assessment services for service users and their families. Interestingly, recent data on waiting times revealed that of patients with an open referral for ASD diagnosis, only 5.6% received an initial appointment within 13 weeks between April 2023 and March 2024 [29]. In addition, the Royal College of Psychiatrists published document CR228 highlighting a commitment to improving mental health services for ASD adults, emphasising the importance of the need for a much broader approach and range of services for ASD adults to have fair access to services and reasonable adjustments to facilitate better outcomes [30].

Typically, diagnostic services employ a triage-based system for referrals [28]. The term refers to a system that sorts patients according to need and the urgency of care [31]. However, the concept of triage can be a source of contention. Here, we affirm that careful, medically informed triage is ethical and necessary. Triage is widespread and standard across several medical settings. There is a strong argument to support the implementation of clinical triage, predominantly in that it is necessary because it involves the best use of resources [32]. Triage provides services with a system to better understand the individual presentation and clinical needs of the service user. It allows the service to gather comprehensive information and enables an informed decision to be made by specialist healthcare professionals regarding the appropriate next step. Triage methods in ASD childhood cohorts have had positive outcomes and provided critical observations [33,34,35,36,37,38,39].

Ultimately, robust assessment pathways are crucial for providing service users with the best possible experience of diagnostic assessment. The aim must be to reduce waiting lists while providing a high-quality assessment that supports established guidance. Research is needed to identify evidence-based, cost-effective models of triage without unnecessary risk to the service user [17,40]. Here, we evaluate the safety and effectiveness of the triage process of an adult ASD Diagnostic Service in the UK using number-needed-to-harm analysis [41,42].

## 2. Materials and Methods

### 2.1. Study Setting and Participants

The study was conducted within a specialist adult Autism Service located in West Yorkshire, UK, between November 2021 and August 2022. Referrals to the Service were primarily made by general practitioners. There are no targets for how many referrals to accept. The acceptance rate depends on the quality of referrals and decisions on a case-by-case basis. The evaluation sample consisted of adults aged 18 years and older, without intellectual disability. During the study period, the service received a total of 985 referrals for autism diagnostic assessment. The Autism Service accepted 185 referrals for triage. The remaining cohort of referrals was deemed clinically inappropriate and was not triaged for assessment. From this cohort, a random sample of 60 cases was selected for evaluation.

Randomisation was conducted weekly, in accordance with the Service’s operational capacity, which allowed for the selection of approximately two to three cases per week. Each case deemed not clinically appropriate during triage was assigned a unique numerical identifier from 1 to *n*, with *n* representing the total number of such cases identified in that week. Random numbers were generated using an online random number generator (Gigacalculator.com: https://www.gigacalculator.com/calculators/random-number-generator.php) within the specified range. Cases corresponding to the randomly generated numbers were selected for inclusion in the study. This process ensured that each case had an equal probability of selection, thereby mitigating selection bias and enhancing the representativeness of the sample.

The selected 60 cases, all initially assessed and triaged as not clinically appropriate for further assessment, were subsequently offered a full diagnostic assessment.

### 2.2. Ethical Considerations

This project was classified as a service improvement activity, and, as such, formal ethical approval was waived by the South West Yorkshire Partnership NHS Foundation Trust (SWYPFT) Research and Development Department. The Caldicott Guardian at SWYPFT authorised access to patient data in adherence to the Caldicott Principles. All data collection activities were conducted by clinicians employed within the Service, and information was extracted exclusively from electronic clinical records. Individual patient consent was not applicable, given the classification of the project as being for service improvement.

### 2.3. Triage Process

The triage process involved two clinicians from a pool of autism specialists, which included consultant psychiatrists, consultant clinical psychologists, and senior advanced practitioners from nursing, occupational therapy, and speech and language therapy disciplines. Each triage session, lasting approximately 20 min, involved a review of both the referral form and the patient’s electronic health record, accessed through the SystmOne system. SystmOne is a secure electronic clinical system utilised across NHS services in the UK, enabling integrated access to patient information, including medical history, allergies, and prescribed medications.

Clinicians adopted an approach specifically intended to minimise confirmation bias. The decision-making framework was structured to assume the potential presence of autism unless sufficient clinical evidence indicated otherwise. This precautionary strategy was designed to reduce the likelihood of false-negative decisions.

The referral form collected demographic information pertaining to the referred individual and the referring clinician, alongside an autism screening questionnaire. It requested specific examples of current difficulties experienced by the individual in relation to social interaction, communication, and behavioural patterns, such as stereotypic or repetitive behaviours and restricted interests. The form further inquired about challenges in educational or occupational settings, difficulties in social relationships, engagement with mental health services, prior diagnoses, and the expectations of the individual regarding the referral. An optional section allowed for contributions from someone familiar with the individual, particularly concerning early childhood behaviours, and space was provided for any additional pertinent information.

The triage panel critically examined all available clinical information from both the referral form and the electronic patient record. A full diagnostic assessment was deemed unnecessary if clear, corroborated evidence from multiple sources demonstrated at least two examples, across two or more DSM-5 core diagnostic domains, that were inconsistent with ASD. For instance, documented evidence of sustained close friendships, effective comprehension of social nuances, active participation in social groups, and an absence of repetitive behaviours would constitute such inconsistency. In such circumstances, the panel concluded that a full assessment was not required. Where referrals were deemed not clinically appropriate, individuals were signposted to alternative services as appropriate, based on identified needs.

## 3. Results

**Figure 1:**
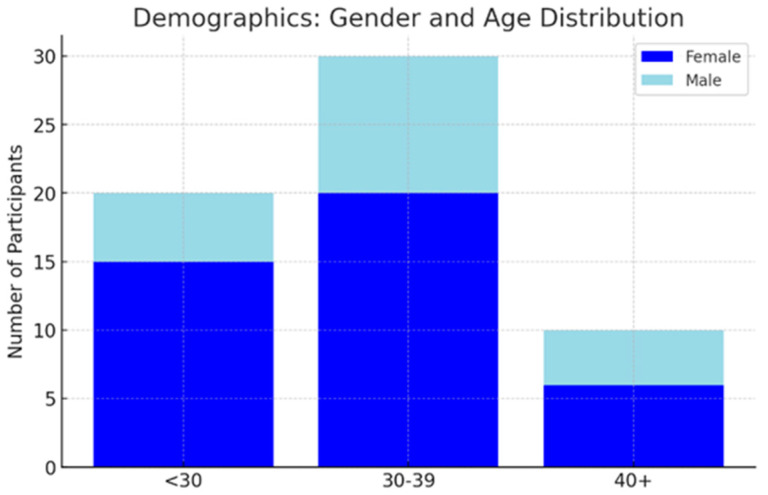
Gender and age distribution.

Among the randomly selected sample of 60 clinically inappropriate cases, 41 were female (69.5%) and 19 were male (32.9%). The age range was 19 to 56 years, with a mean age of 35.5 years (±11.19) (see Figure 1). A statistical comparison of the random sample with the broader cohort demonstrated no significant difference in age distribution (t = −1.66, df ≈ 106, *p* = 0.1003), supporting representativeness in this regard. However, the sex distribution differed significantly between the sample and the broader cohort (Chi-squared = 4.32, *p* = 0.0376), with a higher proportion of females in the random sample.

Full diagnostic assessments were successfully completed for 59 out of the 60 cases, representing a completion rate of 98.3%. One case could not be completed due to the individual relocating during the study period, resulting in an inconclusive outcome.

**Figure 2:**
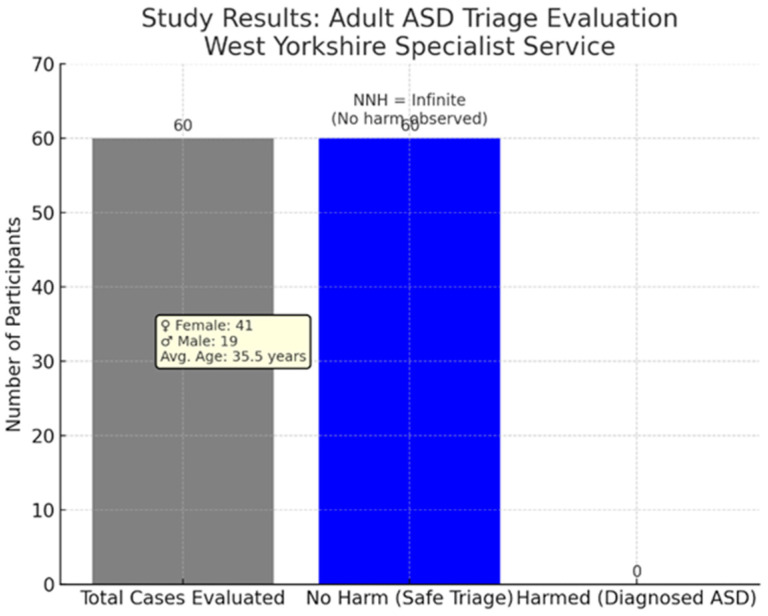
Number needed to harm in triage of adult ASD referral cases.

No clinical diagnoses of ASD were confirmed in the assessed sample. Consequently, the number needed to harm (NNH) was infinite, as there were no cases where the triage process resulted in a missed diagnosis of ASD (see Figure 2). Furthermore, no adverse clinical outcomes were identified during the assessment process, such as distress, disengagement, or safety concerns arising from the offer of full assessment. This outcome supports the conclusion that, within this sample, the triage process functioned effectively without evidence of harm.

The selection of NNH as the primary outcome measure reflects its utility in clinical service improvement contexts, providing a clear metric of the safety profile of the intervention. NNH serves as a counterpart to the number needed to treat (NNT) and offers an accessible and clinically meaningful indicator for interpreting the study findings.

## 4. Discussion

Healthcare for ASD patients is largely unevidenced. Services are under pressure, and the legitimacy of diagnostic pathways and standardised testing is under scrutiny [6]. ASD diagnostic clinics are stretched due to the increased number of referrals, a lack of specialist practitioners, and complex multidisciplinary evaluations. An effective triage strategy is vital for the optimal performance of ASD diagnostic pathways. Here, none of the cases included in the triage evaluation in a UK Adult ASD Diagnostic Service were consistent with ASD. The evaluation demonstrated that triage is safe and necessary for efficient pathway and service utilisation. The approach facilitates decision-making and ensures consistency across cases.

By implementing a robust pathway, services can move toward sustaining diagnostic pathways that work effectively for service users. As previously mentioned, ASD adult populations report experiences of healthcare inequality [4,21], including expressing that their clinical journey is marred with ‘missed opportunities’ and ‘absent support’. A robust diagnostic pathway is imperative in addressing these experiences by providing the most appropriate support in a timely manner.

While this finding highlights an evidence-based approach to adult ASD triage, other factors are pertinent to the wider discussion. For example, triage assessment can be performed within the context of gender-sensitive approaches. Evidence has suggested that both women and those who identify outside of the traditional gender narrative are much more likely to experience delays in accessing diagnostic services and treatment [43]. Burgeoning works in the literature purport that females present ASD markers significantly different from those of males, possibly due to a reduced number of observable behaviours, the debated ideas of the ‘female protective effect’, and a heightened ability to camouflage ASD traits [44]. Taken together with compelling evidence that ASD diagnosis is biassed towards males [45], this is an important consideration as we advance. A limitation of this study was the greater number of females than males in the evaluation, which should be addressed in future evaluations. An additional limitation is the potential introduction of bias into the assessment. We mitigated as much bias as possible by ensuring that most of the assessing clinicians were unaware of the evaluation project; however, some clinicians were privy to this knowledge. In the future, we recommend a double-blind procedure.

The debate surrounding the need to develop evidence-based care pathways will continue, and we trust that this evaluation will go a long way towards extending the evidence in support of robust criteria-based triage procedures within ASD diagnostic services. Other narratives include the consideration of a universal ASD screening programme for children to ease pressures on primary care [38]. Additionally, future directions will inevitably move towards the implementation of artificial intelligence via machine learning; indeed, promising reports concerning this movement exist [46,47,48,49,50,51].

## 5. Conclusions

Our findings support the implementation of criteria-based triage in adult ASD diagnostic pathways, which can significantly enhance service efficiency and the user experience. Ultimately, care pathways must endeavour to employ safe and effective strategies to meet the demand of the service user in the most economically efficient way possible. We argue that an effective adult ASD diagnostic pathway is one that implements fair and robust triage assessment.

## Data Availability

Data sharing is not applicable to this article as no data sets were generated during the current study.

## Abbreviations

The following abbreviations are used in this manuscript:

ASD	autism spectrum disorder
DSM-5	Diagnostic and Statistical Manual of Mental Disorders
GP	general practitioner
NHS	National Health Service
NNH	number needed to harm
NNT	number needed to treat
SWYPFT	South West Yorkshire Partnership NHS Foundation Trust
UK	United Kingdom
